# Decoupling of nutrient stoichiometry in *Suaeda glauca* (Bunge) senesced leaves under salt treatment

**DOI:** 10.3389/fpls.2023.1235443

**Published:** 2023-09-04

**Authors:** Fugui Yang, Shuang Liu, Ma Qian, Donger Wang, Jihui Chen

**Affiliations:** ^1^ College of Animal Science, Guizhou University, Guiyang, China; ^2^ College of Agro-grassland Science, Nanjing Agricultural University, Nanjing, China; ^3^ School of Agriculture and the Environment, Massey University, North Palmerston, New Zealand

**Keywords:** salt treatment, *Suaeda glauca* (Bunge), nutrient stoichiometry, elemental content, saline ecosystems

## Abstract

The stoichiometry of senesced leaves is pivotal in nutrient cycling and can be significantly influenced by soil salinization, a rising global issue threatening the functionality of ecosystems. However, the impacts of soil salinization on senesced leaf stoichiometry are not fully understood. In this study, we conducted a pot experiment with varying soil salt concentrations to examine their influence on the concentrations and stoichiometric ratios of nitrogen (N), phosphorus (P), sodium (Na), potassium (K), calcium (Ca), magnesium (Mg), and zinc (Zn) in the senesced leaves of *Suaeda glauca* (Bunge). Compared to the control group, salt treatments significantly enhanced Na concentration while diminishing the concentrations of K, Ca, Mg, Zn, N, and P. Interestingly, as salinity levels escalated, N concentration maintained stability, whereas P concentration exhibited an increasing trend. Moreover, K, Ca, and Mg significantly declined as salt levels rose. Salt treatments brought about significant changes in stoichiometric ratios, with the N:P, K:Na, N:Na, N:Mg, and Ca : Mg ratios dropping and the N:Ca and N:K ratios rising, illustrating the varying nutrient coupling cycles under different salt conditions. These findings shed light on the plasticity of stoichiometric traits in *S. glauca* senesced leaves in response to soil salinization shifts, which could potentially offer insights into nutrient cycling reactions to soil salinization.

## 1 Introduction

Soil salinization, which affects nearly one billion hectares of land globally, is a critical global issue, posing significant threats to soil function (Herbert et al., 2015; Li et al., 2019; Hassani et al., 2021). This issue is exacerbated by human-induced activities, such as irrigation and land-use change (Flowers et al., 2015; Hassani et al., 2021; Singh, 2021). Soil salinization presents significant challenges to plant growth and development, such as osmotic stress, ion toxicity, and nutrient imbalances, leading to the change in the leaf stoichiometry, a crucial trait regulating nutrient cycling (Sun et al., 2017; Zhang et al., 2023). Furthermore, soil salinization impacts the stoichiometry of senesced leaves (Ge and Xie, 2017; Tong et al., 2021), which significantly influences litter decomposition rates by controlling the activity of soil decomposer, affecting soil nutrient availability and potential for carbon sequestration (Sardans et al., 2012; Hu et al., 2021). Consequently, understanding the effects of soil salinization on the stoichiometry of senesced leaves is crucial for comprehending how soil salinization might modify nutrient cycling in coastal wetland ecosystems.

Nitrogen (N) and phosphorus (P) typically act as limiting nutrients for soil organisms, and senesced leaves contribute a substantial amount of N and P to these organisms (Zechmeister-Boltenstern et al., 2015; Camenzind et al., 2018; Peng et al., 2019). Numerous studies indicate that soil salinization significantly affects the N and P contents and their stoichiometric ratios in plant green leaves. Soil salinization primarily causes an increase in plant N and P contents (Rong et al., 2015; Song et al., 2015; Hurtado et al., 2020; Zhang et al., 2021); however, some studies also report a reduction (Sun et al., 2017; Gong et al., 2020). Therefore, the N:P ratio presents inconsistent results. Thus, these alterations in green leaf stoichiometry may transfer into senesced leaves stoichiometry. However, few studies assert that salt treatment has no significant effects on the N and P contents of senesced leaves and their ratio (Pivničková et al., 2010; Charles et al., 2019). Therefore, more studies need to be conducted to investigate the stoichiometric responses of senesced leaves to soil salinization.

Potassium (K), calcium (Ca), magnesium (Mg), sodium (Na), and zinc (Zn) in senesced leaves also play significant roles in governing essential soil processes, such as decomposition, soil detritus food chains, and storage and turnover of soil carbon (Kaspari et al., 2008; Butler et al., 2019; Zhang et al., 2023). Generally, the salt treatment enhances Na in green leaves while reducing K, Ca, Mg, and Zn contents (Sun et al., 2017; Chrysargyris et al., 2019; Liu et al., 2022). It is vital for plants to maintain a high K:Na ratio to preserve enzymatic activity (Sun et al., 2019), and the Ca : Mg ratio in plant biomass is important for fodder purposes (Klikocka et al., 2018). However, certain studies reveal that salt treatment increases the K:Na ratio in plant leaves but decreases the Ca : Mg ratio (Sun et al., 2017). Although the stoichiometry of green leaves is often reflected in litter composition (Agren and Weih, 2012), this relationship also depends on the traits of the involved elements. Therefore, the responses of elements within green leaves to soil salinization do not consistently indicate the effects on decomposition and related biogeochemical cycles.

*Suaeda glauca*, an annual halophyte widely distributed in saline–alkaline habitats, exhibits high ecological adaptability and salinity resistance (Lu et al., 2021). As an economically valuable halophyte, *S. glauca* is extensively employed for the restoration of polluted or salinized soils (Zhang et al., 2018). Additionally, in China, it is used as fodder for livestock and serves as a foraged food source and medicinal plant for humans (An et al., 2008; Sun et al., 2015). Sun et al. (2017) found that as the salt concentration increased, the Na content in green leaves of *S. glauca* rose, while the contents of P, K, Ca, Mg, and Zn declined. However, the stoichiometric response in senesced leaves to soil salinization remains unclear.

In this study, we aim to explore the impact of different salt levels on the nutrient stoichiometric traits of *S. glauca* senesced leaves. We hypothesize that increasing salt levels will lead to significant changes in the stoichiometric traits of *S. glauca* senesced leaves. Specifically, we expect that as salt levels increase, the Na concentration in senesced leaves will increase significantly, while the concentrations of N, P, K, Ca, Mg, and Zn will decrease significantly. We further hypothesize that these changes in stoichiometry in senescent leaves are primarily driven by changes in stoichiometry in green leaves. This investigation will provide valuable insights into the stoichiometry response of senesced leaves to salt treatment and contribute to a better understanding of the effects of increasing soil salinization on nutrient cycling in saline ecosystems.

## 2 Material and methods

### 2.1 Plant materials

At the end of April, *S. glauca* seedlings, approximately 6 cm tall, were collected from the Yangtze River Delta seashore (located in Jiangsu Yancheng Wetland National Nature Reserve, 33°00.688N, 120°50.755E). Following collection, these seedlings were transferred into 54 plastic pots (30 cm in diameter, 35 cm in depth), with three plants per pot serving as a single replicate. Each pot was filled with perlite. The pots were kept under greenhouse conditions, with a maintained relative humidity of 70%. The day/night temperature range was kept between 20°C–35°C and 15°C–25°C, and the plants were exposed to natural light. A half-strength Hoagland solution was used for daily watering.

### 2.2 Experimental design

The pots were arranged into six groups, each containing nine replicates. Each group was subjected to one of six different sea salt treatment concentrations (0.001 mol/L, 0.05 mol/L, 0.1 mol/L, 0.2 mol/L, 0.4 mol/L, and 0.8 mol/L). Following 16 days of treatment, the perlite was consistently moistened with a modified half-strength Hoagland nutrient solution, incorporating the appropriate sea salt concentrations. The water level was adjusted daily to compensate for evaporation and prevent excessive salt accumulation in the pots. The salt solutions were refreshed every 3 days. For control purposes and to minimize any environmental impact on plant growth, the pots were rearranged every week.

#### 2.2.1 Sampling and chemical analysis

In late October, the senesced leaves of the *S. glauca* were collected. The samples were oven-dried for 72 h at 65°C, weighed, and then ground in 20-inch mesh in Wiley Mill. The samples were stored in a cool, dry place for subsequent chemical analysis. The total N concentrations in the litter (mg/g) were determined in 0.01-g plant tissue subsamples and analyzed with an elemental analyzer (Vario EL III, Elementar, Langenselbold, Germany). The concentrations of P, K, Ca, Mg, Zn, and Na (mg/g) were measured with an inductively coupled plasma (ICP) emission spectrometer (Iris Advantage 1000, New York, NY, USA), following the digestion of subsamples in a blend of trace metal-grade H_2_SO_4_–HClO_4_.

#### 2.2.2 Statistical analysis

A one-way ANOVA was used to assess the effects of salt treatment on element concentrations and element mass ratios in the senesced leaves. Tukey’s multiple comparison test (*p* < 0.05) was performed to identify significant differences among treatments. Pearson’s correlation coefficients were calculated to analyze the relationships between different variables in the senesced leaves, including the concentrations and mass ratios of the elements. All data analyses were conducted using SPSS20.0 (SPSS 20.0 for Windows, USA).

## 3 Results

### 3.1 Concentration of mineral elements in senesced leaves

**Figure 1:**
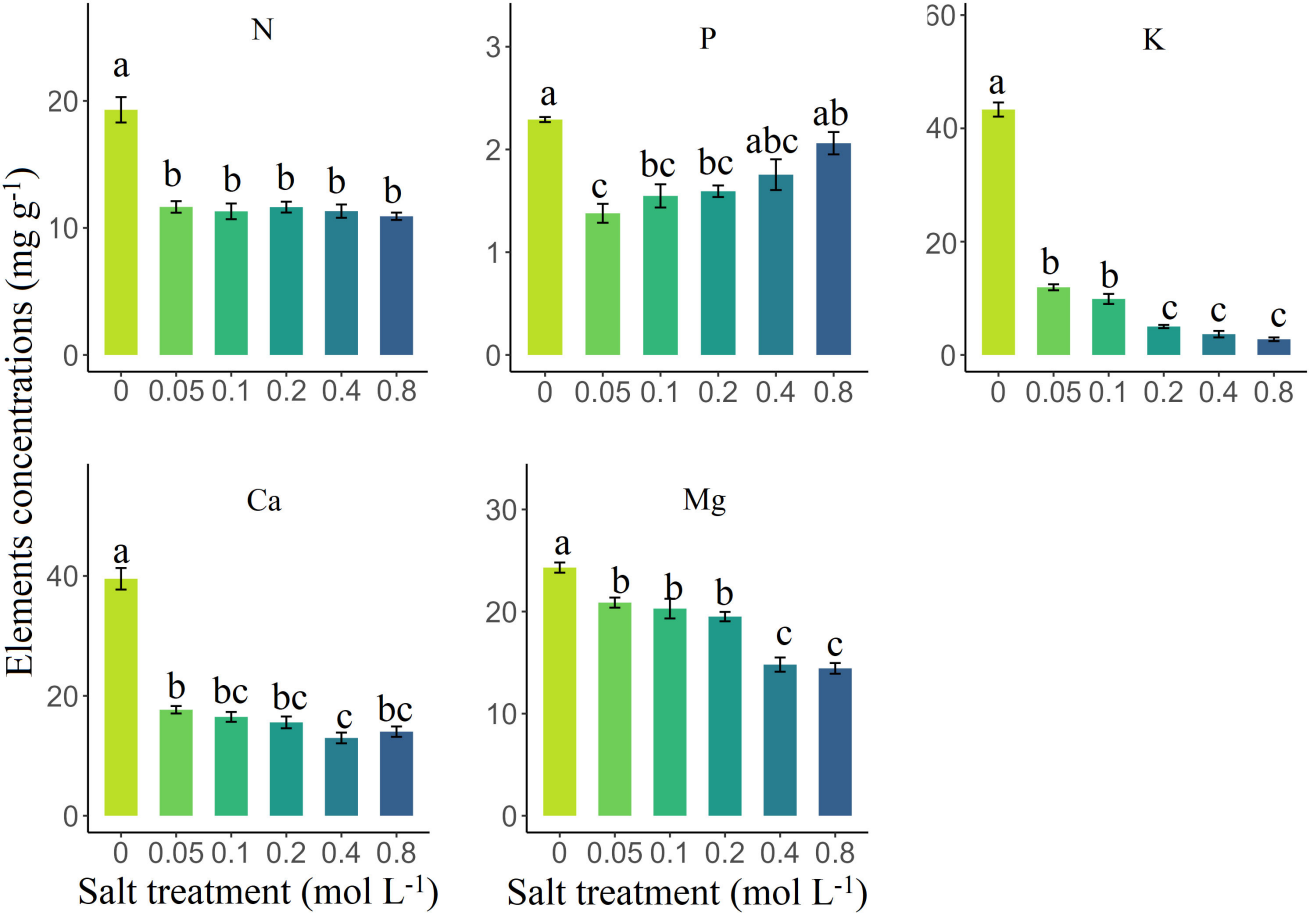
The influence of salt treatment on the N, P, K, Ca, and Mg concentrations (mg/g) in senesced leaves of *Suaeda glauca*. Different lowercase letters represent significant differences (Tukey’s multiple comparison, *p* < 0.05).

**Figure 2:**
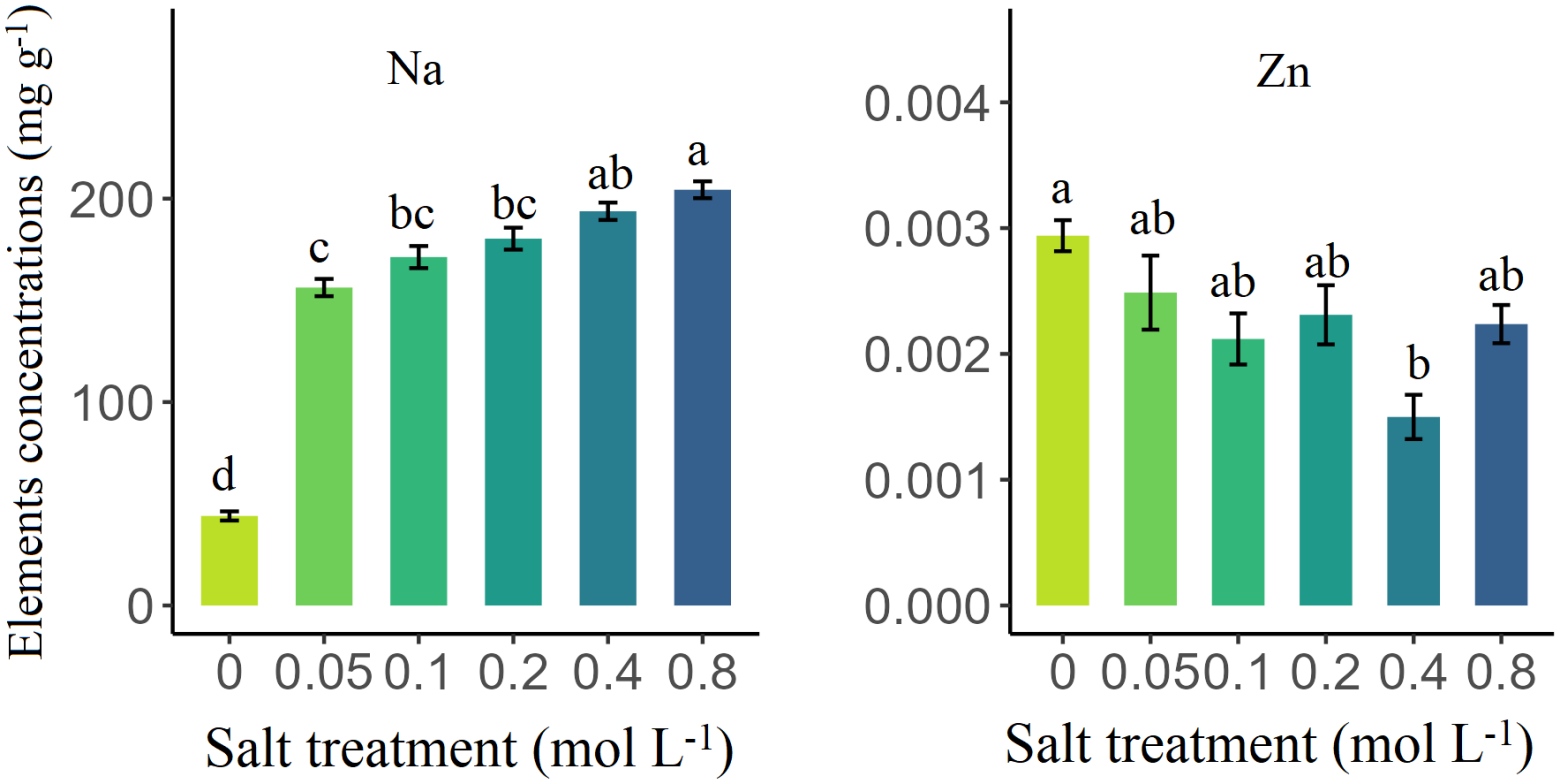
The influence of salt treatment on the Na and Zn concentrations (mg/g) in senesced leaves of *Suaeda glauca*. Different lowercase letters represent significant differences (Tukey’s multiple comparison, *p* < 0.05).

Salt treatment significantly affected N, P, K, Ca, Mg, Zn, and Na concentrations in senesced leaves of *S. glauca* (*p* < 0.05) (**Table 1**). Compared to the control group, the concentrations of N, P, K, Ca, and Mg under salt treatment decreased by 41.02%, 27.74%, 84.37%, 61.08%, and 25.59%, respectively. Conversely, the Na increased by 310.11% under salt treatment, while Zn decreased by 27.78%. Furthermore, the contents of P and Na increased significantly across all salt treatments (*p* < 0.05), reaching maximum values of 2.06 mg/g and 204.37 mg/g at a concentration of 0.8 mol/L salt concentration, respectively. However, the concentrations of K, Ca, Mg, and Zn decreased significantly, reaching their lowest values of 2.77 mg/g, 12.98 mg/g, 14.43 mg/g, and 0.0015 mg/g, respectively (*p* < 0.05). Notably, the N content did not change significantly change among all salt treatments (*p* > 0.05) (**Figures 1**, **2**).

**Table 1:** F values from one-way ANOVA with salt treatment for N, P, K, Ca, Mg, Na, and Zn concentrations (mg/g) and mass ratios in leaves of *Suaeda glauca*.

	N	P	K	Ca	Mg	Na	Zn	K/Na	Ca/Mg	N/P	N/K	N/Ca	N/Mg	N/Na	N/Zn
Salt treatment	19.99***	7.50***	343.40***	65.77***	25.59***	83.18***	3.56*	123.00***	21.47***	9.99***	23.40***	3.51*	7.09***	636.42***	2.49

Asterisks denote significance: *p < 0.05; **p < 0.01; ***p < 0.001.

The concentrations of different elements in the senesced leaves of *S. glauca* varied considerably (**Table 2**). The coefficient of variation (CV) was the largest for the K content (110.93%), followed by Ca (41.50%), Zn (30.44%), Na (25.12%), P (22.13%), N (21.16%), and Mg (19.08%).

**Table 2:** Coefficient of variation (%) of N, P, K, Ca, Mg, Na, and Zn concentrations (mg/g) and mass ratios in leaves of *Suaeda glauca* along salt concentrations.

	N	P	K	Ca	Mg	Na	Zn	K/Na	Ca/Mg	N/P	N/K	N/Ca	N/Mg	N/Na	N/Zn
CV	21.16	22.13	110.93	41.50	19.08	25.12	30.44	225.47	27.06	18.87	66.88	21.05	20.81	110.30	33.87

**Figure 3:**
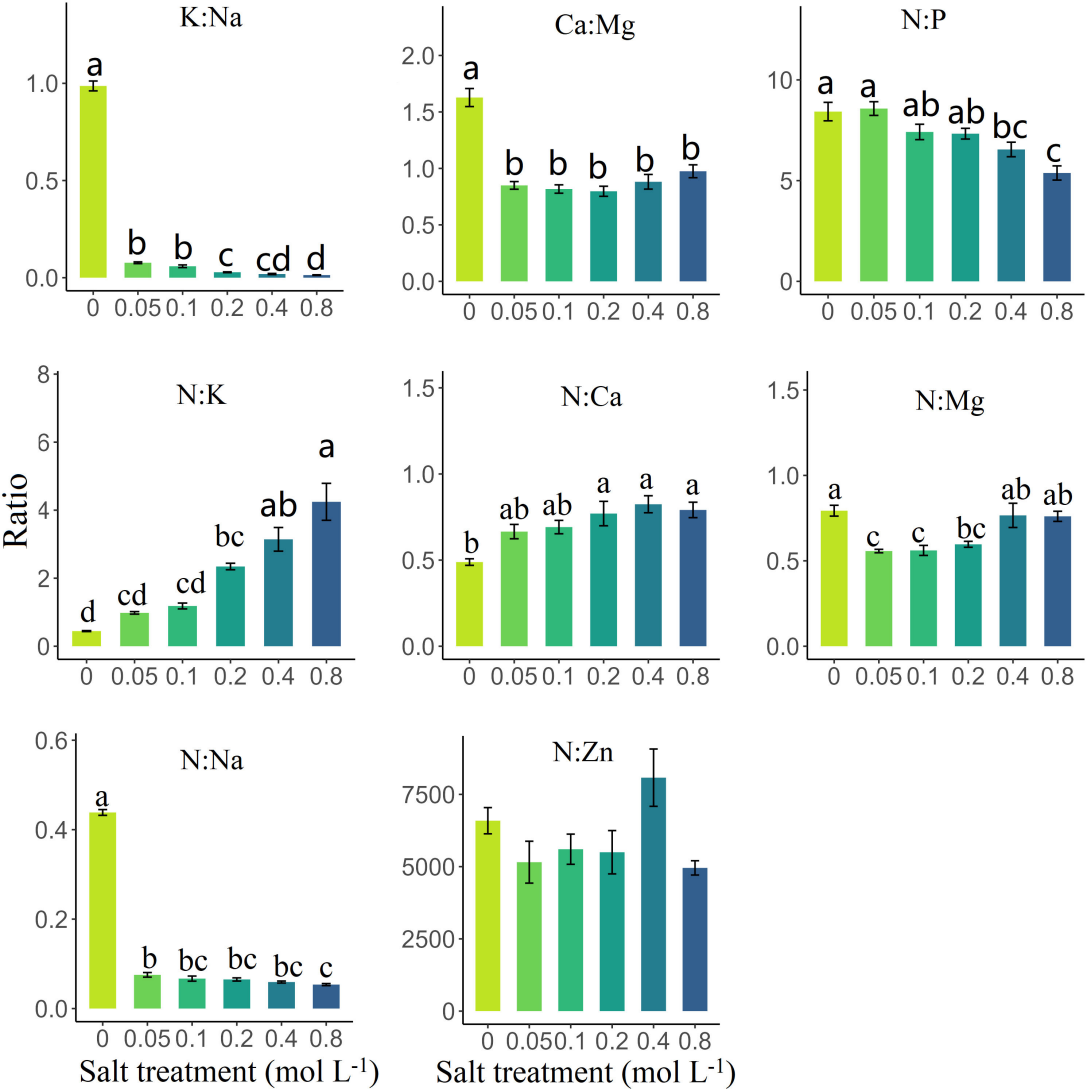
The influence of salt treatment on the elements mass ratios in senesced leaves of *Suaeda glauca*. Different lowercase letters represent significant differences (Tukey’s multiple comparison, *p* < 0.05).

Mineral element ratios in senesced leaves salt treatment significantly influenced the element ratios in senesced leaves of *S. glauca* (*p* < 0.05), except for the N:Zn ratio (**Table 1**). The K:Na ratio decreased significantly at low salt concentrations (0.05 mol/L) and continued to decrease more gradually with increased salt treatment (*p* < 0.05) (**Figure 3**). The N:Na ratio exhibited a similar decreasing trend (**Figure 3**). The Ca : Mg ratio in leaves reduced significantly by 47.10% in salt-treated leaves compared to the control (*p* < 0.05), while other salt treatments showed no significant differences. Interestingly, the N:Mg ratio initially increased with increasing salt concentration and then decreased (**Figure 3**). The N:P ratio remained stable under low and medium salt concentrations (0.05 mol/L to 0.2 mol/L) but fell significantly under high salt concentrations (≥0.4 mol/L) compared to the control group (*p* < 0.05) (**Figure 3**). In contrast, the N:K and N:Ca ratios significantly increased along with rising salt concentrations (*p* < 0.05) (**Figure 3**). The CV was the highest for K:Na (225.47%) and the lowest for N:P (18.87%), with intermediate values observed for N:Na, N:K, N:Zn, Ca : Mg, N:Ca, and N:Mg (**Table 2**).

### 3.2 Relationship between different mineral elements and ratios

**Figure 4:**
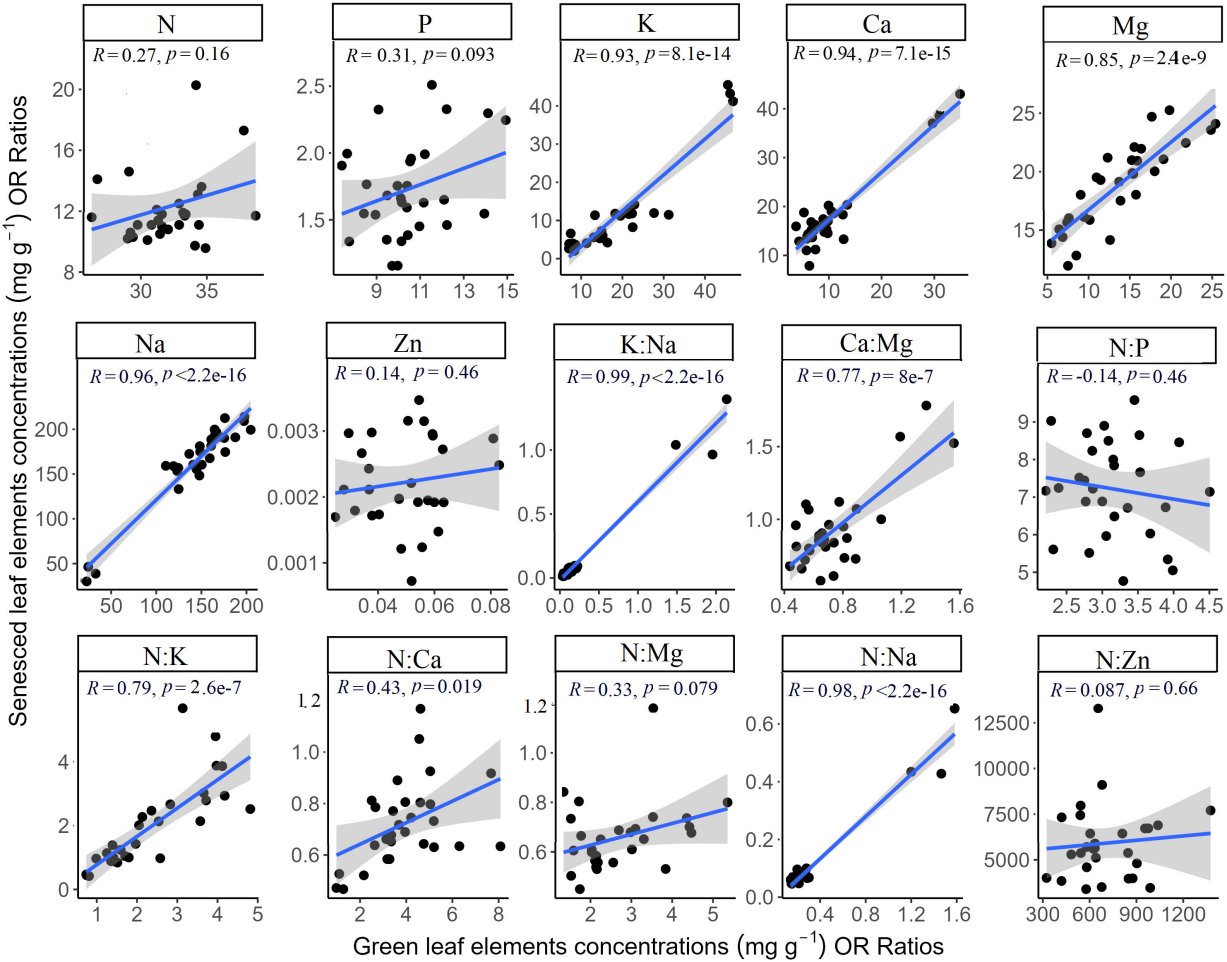
The relationship between green leaves stoichiometry and senesced leaves stoichiometry.

A significant positive correlation was found between the K, Ca, Mg, and Na concentrations, as well as their K:Na, Ca : Mg, N:K, N:Ca, N:Mg, and N:Na ratios in the senesced and green leaves (*p* < 0.05) (**Figure 4**). No significant correlations were identified for other stoichiometric traits between the senesced leaves and green leaves (*p* > 0.05) (**Figure 4**).

Additionally, Pearson’s correlation analysis revealed complex relationships between elements in senesced leaves (**Table 3**). N displayed a significant positive correlation with all elements except Na (R^2^ = −0.86, *p* < 0.05). Similarly, K, Ca, and Mg correlated positively, but negatively with Na (*p* < 0.05). P and Zn displayed selective correlations with N, K, Ca, and other elements, without significant associations with certain elements. Elemental ratios, such as K:Na, Ca : Mg, and N:P, correlated strongly with K, Ca, and P. N:K, N:Ca, and N:Mg showed negative associations with K, Ca, and Mg, whereas N:Na and N:Zn exhibited mixed correlations.

**Table 3:** Correlation analysis between different element contents and ecological stoichiometric ratio.

	P	K	Ca	Mg	Na	Zn	K/Na	Ca/Mg	N/P	N/K	N/Ca	N/Mg	N/Na	N/Zn
N	0.55***	0.87***	0.85***	0.55***	−0.86***	0.40*	0.87***	0.71***	0.30*	−0.35*	−0.19	0.44**	0.91***	0.12
P		0.32*	0.33*	−0.19	−0.24	0.09	0.43**	0.56***	−0.61***	0.21	0.19	0.80***	0.46**	0.13
K			0.96***	0.70***	−0.97***	0.42*	0.96***	0.76***	0.44**	−0.61***	−0.54***	0.13	0.96***	0.06
Ca				0.64***	−0.95***	0.50**	0.96***	0.86***	0.39*	−0.50**	−0.66***	0.15	0.96***	0.00
Mg					−0.77***	0.40*	0.56***	0.17	0.68***	−0.73***	−0.49**	−0.48**	0.56***	−0.11
Na						−0.45**	−0.93***	−0.71***	−0.50**	0.60***	0.57***	−0.04	−0.93***	−0.07
Zn							0.39*	0.39*	0.30*	−0.21	−0.40*	−0.01	0.40*	−0.77***
K/Na								0.84***	0.31*	−0.43*	−0.50**	0.26*	0.99***	0.09
Ca/Mg									0.04	−0.18	−0.55**	0.51**	0.84***	0.06
N/P										−0.58***	−0.35*	−0.43**	0.31*	−0.09
N/K											0.48**	0.35*	−0.40*	−0.02
N/Ca												0.39*	−0.46**	0.19
N/Mg													0.30*	0.21
N/Na														0.10

Asterisks denote significance: *p < 0.05; **p < 0.01; ***p < 0.001.

## 4 Discussion

### 4.1 Effects of salt treatment on mineral elements contents in leaves of *S. glauca*

In accordance with our prior observations on the stoichiometry of *S. glauca* green leaves’ response to salt treatments (Sun et al., 2017), the Na content in senesced leaves increased following salt treatment. A strong correlation was found between the Na content of senescent and green leaves, suggesting that the Na content in green leaves determines changes in Na concentration in senescent leaves (**Figure 4**). As a halophyte, *S. glauca* can tolerate high salt concentrations, leading to an increase in leaf Na content as the salt concentration rises (Sun et al., 2017; Chrysargyris et al., 2019). This shows that *S. glauca* can accumulate Na to maintain osmotic balance and alleviate the harmful effects of high salinity (Arif et al., 2020). One possible mechanism may be that high salt concentration in the soil triggers osmotic stress in the plant, causing an upregulation of Na transporters in the plant’s roots. Consequently, the plant can absorb more sodium from the soil, helping to maintain cell osmotic balance.

However, salt treatments led to a decrease of K, Ca, Mg, and Zn concentrations in senesced leaves, with the concentrations of these elements continuously declining as the salt concentration increased. Given the strong correlation between K, Ca, and Mg concentrations in senescent and green leaves (**Figure 4**), these shifts are likely due to changes in these elements within the green leaves. Na accumulation inhibits the absorption of K, Ca, and Mg due to their similar chemical properties (Isayenkov and Maathuis, 2019). This interpretation is supported by the negative relationship between Na and other elements (K, Ca, Mg, and Zn) in *S. glauca* leaves (**Table 3**). Therefore, salt treatment resulted in a significant decrease in K, Ca, and Mg concentrations in senescent leaves, and the variation of K concentration in senesced leaves under salt treatment was greater than that of Ca and Mg (respectively, 110.93%, 41.50%, and 19.08%). This is due to Na^+^ and K^+^ having similar hydration radii and can exhibit a strong antagonism under salt treatment (Li et al., 2010). As a result, high Na^+^ concentrations compete with K^+^ for uptake sites, limiting K^+^ absorption and leading to lower leaf K content (Rejili et al., 2007). Salinity influences the osmotic potential of the soil, hindering plant roots from absorbing other mineral nutrients (Sheldon et al., 2017), such as Zn in this study.

N and P are two critical elements contributing to plant vegetative development, reproduction, and plant–soil trophic interactions (Sardans et al., 2017; Mao et al., 2018). In our study, we found that low salt treatment (0.05 mol/L) diminished N and P levels in plant leaves, which is consistent with previous research (Gong et al., 2020). Several factors could contribute to this observation. First, the biomass dilution effect might be responsible, evidenced by the increase in biomass (Sun et al., 2017). Furthermore, the significant negative correlation between N content and Na content in senescent leaves implies that an increase in Na content under salt treatment can inhibit plant N absorption. Finally, the enhancement of N and P resorption due to salt treatment might also provide a partial explanation, as N and P resorption efficiency significantly increased with soil salinity (Luo et al., 2021). Interestingly, as salt treatment intensified, plant N remained consistent, whereas P exhibited a rising trend, with no significant correlation found between senescent leaves and green leaves. This could be because under saline conditions, plants synthesize more cellular antioxidant and reactive oxygen species (ROS)-scavenging enzymes than under control conditions (Cabot et al., 2014). Given that N is an integral part of all enzymes (Khan et al., 2014; Mansour and Hassan, 2022), plants may maintain stable N content to adapt to salt stress. A similar result was also found in a coastal wetland field investigation (Rong et al., 2015). The distinctive trend of P along salt concentration could be attributed to a decrease in seed production, which subsequently reduces the transfer of P from vegetative organs to seeds—seeds usually contain higher P levels than vegetative organs (Güsewell, 2004; Zhou et al., 2016).

Overall, our findings suggest that salt treatment significantly impacts the stoichiometry of senescent leaves in *S. glauca*. We observed significant associations between changes in Na, K, Ca, Mg, and Zn contents in senescent leaves and alterations in green leaf stoichiometry, which partially supports the concept of green leaf control over litter stoichiometry. Also, changes in N and P contents could potentially be linked to the nutrient reabsorption process.

### 4.2 Effects of salt treatment on the stoichiometry ratios of *S. glauca*

Alterations in salt treatment brought about significant changes in the multi-element stoichiometric ratios of senesced leaves, especially concerning the ratios of Na to K (Song et al., 2015). This phenomenon was also observed in green leaves (Sun et al., 2017), reflecting a higher rate of Na absorption relative to K. The N:P ratio of senesced leaves, a key factor in controlling the litter decomposition by soil microorganisms (Mooshammer et al., 2012), remained unaltered under low salt treatments but exhibited a significant decrease at high salinity levels. There could be several reasons for this. First, low salt treatments promote the growth of halophytes, enabling these plants to maintain homeostasis of the N:P ratio (Minden and Kleyer, 2014; Zhang et al., 2023). Second, under conditions of high salt treatment, plants need to adapt to the salt stress, potentially reducing reproduction (as discussed above), leading to lower N:P ratios. Our correlation analysis also shows that the N:P ratio correlates more strongly with P than with N. Furthermore, the ratio of N to other elements in senesced leaves was significantly affected by salt treatment. As the intensity of salt treatment increased, the N:Na ratio in senesced leaves decreased since the N concentration initially dropped and then stabilized, while the Na concentration continually increased. Conversely, given that salt treatment substantially inhibited the uptake of K, Ca, and Mg, the ratios of N to these three elements all rose as the salt concentration increased.

The Ca : Mg ratio in plants has a substantial impact on the health of the organisms that consume them (Ostrowska and Porębska, 2017). Our results found that low-salt treatment decreased the Ca : Mg ratio of senesced leaves, while the ratio remained constant despite increasing salt concentrations. This observation may be attributable to the fact that the rate of decrease in Ca concentration caused by salt treatment surpasses the rate of decrease in Mg concentration.

### 4.3 Implications for nutrient cycle

The release of nutrients from plant litter to the soil significantly influences global carbon and nutrient cycling, and the stoichiometry of this plant litter acts as a key controlling factor in litter decomposition (Manzoni et al., 2008). An increase in Na concentration in senesced leaves can stimulate microorganism activity and accelerate the decomposition of these leaves (Ji et al., 2020). The decrease of N:P ratios under high salt conditions may cause N limitation on the decomposer, decoupling N and P cycling, with potential consequences for ecosystem productivity and stability (Wang and Yu, 2008). This impact could be further aggravated by climate change, such as rising temperatures. Additionally, the differential responses of various nutrient ratios to salt treatment could affect the competitive interactions between *S. glauca* and other plant species in these habitats. This interaction could, in turn, shape the composition and structure of saline ecosystems, necessitating further study.

## 5 Conclusion

In conclusion, our study reveals the effects of salt treatment on stoichiometry in *S. glauca* senesced leaves. The findings emphasize the complex reactions of plants to saline conditions and provide key insights into plant stoichiometric traits. Moreover, this research offers insights into the potential effects of global environmental changes on the functioning of saline ecosystems. Further exploration is needed to understand underlying mechanisms and potential management strategies to mitigate the negative impacts of salinization on nutrient cycling.

## Data availability statement

The raw data supporting the conclusions of this article will be made available by the authors, without undue reservation.

## Author contributions

FY and JC contributed to the conception and design of the study. DW organized the database. JC performed the statistical analysis. FY wrote the first draft of the manuscript. SL and MQ wrote sections of the manuscript. All authors contributed to the manuscript revision and read and approved the submitted version.
